# Assessment of color preference, purchase intention and sexual attractiveness of lipstick colors under multiple lighting conditions

**DOI:** 10.3389/fnins.2023.1280270

**Published:** 2023-11-14

**Authors:** Baolin Tian, Hanwen Gong, Zhiyu Chen, Xuan Yu, Michael R. Pointer, Jie Yu, Feng Yu, Qiang Liu

**Affiliations:** ^1^Department of Psychology, College of Philosophy, Wuhan University, Wuhan, China; ^2^Colour Technology Research Group, School of Design, University of Leeds, Leeds, United Kingdom; ^3^Joint Laboratory of Light Quality and Colour Vision, Wuhan University and OPPLE, Wuhan, China; ^4^Opple Lighting Co., Ltd., Shanghai, China; ^5^Laboratory of Lighting Technology, Technische Universität Darmstadt, Darmstadt, Germany

**Keywords:** lipstick color, color perception, gender difference, red effect, correlated color temperature

## Abstract

Lipstick is one of the most commonly used cosmetics, which is closely associated with female attractiveness and influences people’s perception and behavior. This study aimed to investigate the impact of light sources, lipstick colors, as well as gender on the subjective assessment of lipstick color products from the prospective of color preference, purchase intention and sexual attractiveness. The correlation between color preference evaluations when applying lipstick on lips and on forearms was also explored. Sixty participants completed their visual assessment of 15 lipsticks worn by 3 models under 5 light sources, with uniformly sampled correlated color temperature (CCT) values ranging from 2,500 K to 6,500 K. The results indicated that the light source significantly influenced color preference and purchase intention, while lipstick color significantly impacted on sexual attractiveness. The interactions between gender and other factors were also observed and are discussed. Compared to men, women were found to be more sensitive to different light sources and hold different attitudes toward different lipstick colors under different CCTs. Interestingly, no significant correlation was found between lipstick color preference ratings on the lips and forearm, which conflicted with the commonly recognized way of lipstick color selection. These findings should contribute to a deeper understanding of the consumer attitude toward lipstick colors and provide a useful reference for lighting design in situations where cosmetics are specified, manufactured, retailed and generally used, both professionally and in the home.

## 1. Introduction

Throughout history, colors have been argued to have important influences on human mind and behavior (Maule et al., 2023), with one notable aspect being attractiveness (Elliot and Maier, 2012). Previous studies have demonstrated that black and red have an elevating effect on perceived attractiveness (Roberts et al., 2010), while other colors are much less pronounced. Compared to the black which gives prominence to fashion (Pazda et al., 2013), the color red has more complications of content (Elliot and Maier, 2014) and has garnered wide research attention. Many studies have demonstrated that red can enhance men’s perception of women’s attractiveness. Women wearing red shirts were considered more attractive than those wearing blue, green or white shirts (Guéguen and Jacob, 2012a; Pazda et al., 2012). Their pictures were also perceived as more attractive and sexually desirable by manipulating a red background (Elliot and Niesta, 2008). Guéguen et al. have also found that women wearing red clothing, compared to other colors, were more likely to be picked up by male drivers, but not by female drivers (Guéguen, 2012a). This highlights the unique signaling role of red to men. In biology, the display of red on the face, chest, or genitals of non-human female primates during their fertility phase represents a sexual signal designed to attract mates (Dixson, 1983; Deschner et al., 2004; Setchell and Wickings, 2004; Barelli et al., 2007). Similarly, during ovulation, human females may experience reddened skin on their face or body due to increased blood flow velocity (Roberts et al., 2004; Lynn et al., 2007), and are more likely to be sexually aroused (Bullivant et al., 2004), displaying the red flushes of sexual excitation (Katchadourian and Lunde, 1972). After the long process of evolution, men, like other male animals, may have come to subconsciously associate the color red with sexual signals (Elliot and Niesta, 2008; Pazda et al., 2013).

The lipstick, one of the most popular cosmetics, with red being a universally used color, is closely associated with this red effect. In 2012, Guéguen conducted a study that found a correlation between women wearing red lipstick and being approached by men in a bar (Guéguen, 2012b). Furthermore, in another experiment where female waitresses changed the color of their lipstick as the variable in a restaurant, it was observed that waitresses wearing red lipstick received more tips from male customers (Guéguen and Jacob, 2012b). Some researchers speculated that the use of red lipstick may serve as an attempt to emulate the vascularization present during ovulation and sexual excitation (Low, 1979). These findings support the notion that red lipstick is strongly linked to women’s attractiveness, with sexual appeal being a key aspect due to the association between the color red and sex. Apart from sexual appeal, lipstick can darken the lip color, increase the brightness contrast between lips and the surrounding skin (Russell, 2009) and make women’s faces appear younger and increase femininity (Porcheron et al., 2013), so as to enhance the sexual dimorphism and their attractiveness (Stephen and McKeegan, 2010; Guéguen and Jacob, 2012b). Additionally, lip color can provide insight into an individual’s health status, such as cardiopulmonary function and blood oxygen concentration, as blue lips indicate cyanosis, which is a condition characterized by insufficient blood oxygen levels linked to cardiac and respiratory illness (Ponsonby et al., 1997). Thus, lipstick can conceal or give prominence to people’s psychological states and physiological and convey various information by changing the lip color.

As mentioned previously, the role of lipstick is closely linked to the color red. However, it is worth noting that within this color range, there are countless variations of red with subtle or remarkable color distinctions. Prior studies have explored how different lipstick colors, such as red, pink, and brown, affect women’s attractiveness (Guéguen, 2012b; Guéguen and Jacob, 2012b). Nevertheless, there is still limited understanding on the effects of moderate color variations within the primary lipstick shades, like true red, orange red and cameo red, despite they are the most prevalent and widely used colors in everyday cosmetic routines. Therefore, the current study aimed to investigate whether these popular shades with moderate differences have an impact on people’s perception of lip color and how they affect the subjective evaluations.

At the same time, in practical applications, the availability of multiple lipstick colors necessitates cosmetic-counter trials for individuals to determine suitability for specific use. In this process, lighting plays a crucial role. Previous studies have demonstrated that lighting significantly impacts the color appearance of objects and thus affects many visual attributes such as color fidelity (Nickerson and Jerome, 1965; Davis and Ohno, 2010), color preference (Bodrogi et al., 2015; Huang et al., 2017, 2019a,b), color naturalness (Jost-Boissard et al., 2009, 2014), color vividness (Khanh and Bodrogi, 2016; Khanh et al., 2016a,b) and color discrimination (Jiang et al., 2015). Furthermore, as proposed by former researchers, both lighting application (Kees and Christoph, 2016; Tang and Teunissen, 2018) and experimental objects (Jost-Boissard et al., 2009, 2014) affect visual color perception. These findings imply that general research conclusions on the color quality of lighting may not satisfy all individual needs, necessitating more focused research for specific applications (Teunissen, 2019; Weirich et al., 2022). The perceived color of red cosmetic products is relatively special, as it is more vivid and saturated, which is influenced by both the spectral power distribution (SPD) of the light source and the spectral reflectance of the object. These facts emphasize the importance of illumination when choosing makeup products. However, few studies have examined the influence of lighting on observers’ perception and evaluation of the red cosmetics.

In addition to applying lipstick directly to the lips, another commonly-used method for assessing its color is to apply lipstick to the inner sides of the forearms, and thus display multiple colors simultaneously. This method is frequently used in real purchasing situations, such as online product displays, sales presentation, and consumer trials. While this method has been widely accepted by the public, there is no scientific evidence to support the consistency of color appearance between lipstick stripes on the forearms and those colors on the lips. The validity of using this method to evaluate the preference level for different lipstick colors is even more questionable. Due to the complexity of color perception, the effect of different presentation styles was worth being explored and we suspected that the method of presenting lipstick color may not be as effective as it seems, and it might mislead consumers.

Finally, it is worth noting that, despite the common association of lipstick with females, as mentioned earlier, the association between red and sexuality plays a role when men perceive women’s attractiveness. As a result, differences in gender-based color perception and assessment may exist regarding lipsticks. Additionally, differences in color vision between men and women have been extensively reported in studies from multiple disciplines, including genetics (Vanston and Strother, 2017), neuroscience (Palmer et al., 2013; Alfano et al., 2023; Young et al., 2023), ophthalmology (Panorgias et al., 2010), and biology (Hurlbert and Ling, 2007; Schwarzkopf et al., 2011). For instance, evidence related to gender differences was found in androgen receptors, estrogen, and genes on the X chromosome (Neitz and Neitz, 2011; Vanston and Strother, 2017). Therefore, we sought to explore potential sex-related variations in lipstick color perception.

In this study, three response scales were used to evaluate the lipstick colors, including color preference, purchase intention, and sexual attractiveness. Color preference represents a general, subjective evaluation of overall the esthetic feelings toward the color of an object; purchase intention is closely related to the product attributes of lipsticks (i.e., the consumers’ desire to buy) while sexual attractiveness is included in order to further explore the previously mentioned red effect for both sexes and to investigate how males and females respond when asked about the extent of “sexual attractiveness” of a female wearing lipstick. The selection of these three dimensions considered both the evaluation aspect of general subjective evaluation experiments and the specific characteristics of lipstick. Some previous studies have researched the color of cosmetics through Internet survey (Mulhern et al., 2003; Pazda et al., 2023), which may affect the rendering effects owing to the absence of rigorous control of the color stimuli (Elliot, 2018). Other studies have investigated the effects of different lighting on red cosmetic products in a lighting booth (Khanh and Bodrogi, 2016), which is detached from actual usage scenarios where cosmetic products are actually applied to the skin. Our experiment overcame these limitations by adopting a more realistic and valid approach of presenting lipstick colors in real scenes, with a comprehensive consideration of the mechanism of color appearance (Li et al., 2017). By conducting this research, the effects of lipstick colors, light source properties, gender, and presentation styles on lipstick color assessment were comprehensively investigated.

## 2. Method

### 2.1. Participants

A panel of 60 observers, 30 males and 30 females, participated in the study. All participants were students of Wuhan University and had passed the Ishihara Color Vision Test (Ishihara, 1960). Prior to participation, all participants were pre-screened to ensure they were heterosexuals, as sexual attraction was included as a research dimension. The age range of the participants was 16 to 26 years, with a mean age of 21.2 years and a standard deviation of 2.3 years. Note that there is one subject with an age of 16 and consent has been acquired by the subject’s parents. The research purpose was not disclosed to participants before the experiment. Following completion of the study, participants received a compensation of 50 CNY (Chinese Yuan). Prior to the experiment, all participants were aware of and agreed to all procedures in the experiment. The Institutional Review Board (IRB) of Wuhan University approved the experiment involving human subjects.

### 2.2. Lighting condition

**Figure 1:**
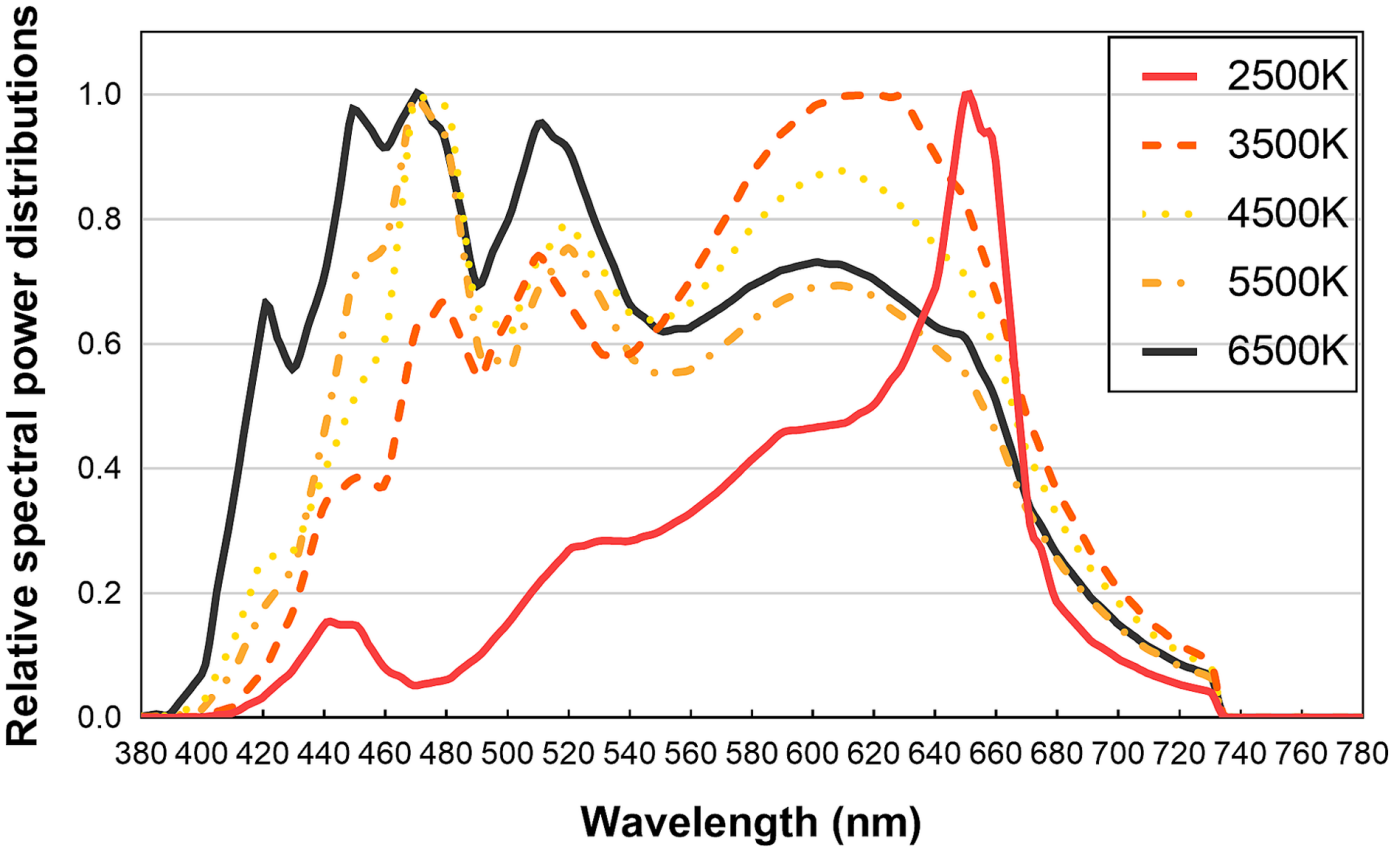
Relative spectral power distributions of the experimental light sources.

The experiment was conducted in a dark room equipped with two LED (light-emitting diode) lamps positioned at a 45° angle to the model’s central axis. The model was seated on a height- adjustable chair in a fixed position, while the LED lamps were situated 30 cm away from the model’s face and set at the same height as the model’s face to ensure uniform illumination. To prevent interference from other colored objects, a gray background was used and observers were also asked to wear gray cloths. Five light sources were generated by the spectrally tunable smart lighting system, named LED cube from Changzhou Thouslite Ltd., with correlated color temperatures (CCT) of 2,500 K, 3,500 K, 4,500 K, 5,500 K, and 6,500 K. The Duv values (distance between the chromaticity coordinates of the light source and the blackbody locus) approximately equal to 0, and the color rendering indexes (Ra) were between 90 and 95. The illuminance level on the model’s lips was approximately 1,100 lx (unit of illuminance, which is the total luminous flux incident on a surface, per unit area). The relative SPD of each light source was measured using an X-Rite i1 Pro2 (X-RITE, 2020) spectrophotometer, as shown in Figure 1. The colorimetric parameters of the experimental lights are presented in Table 1.

**Table 1:** The colorimetric properties and typical color quality metric values of the experimental light sources.

ID	2,500 K	3,500 K	4,500 K	5,500 K	6,500 K
Measured CCT (K)	2,542	3,503	4,503	5,489	6,516
x	0.47	0.41	0.36	0.33	0.31
y	0.41	0.39	0.36	0.34	0.32
u’	0.27	0.24	0.22	0.21	0.20
v’	0.53	0.51	0.49	0.48	0.47
Duv	0.000	0.000	0.000	0.000	0.000
CRI(Ra)	95	91	90	90	93
GAI	48	66	82	93	102
Qa(v9.0.3)	92	91	91	93	95
Qf(v9.0.3)	91	91	91	93	94
Qg(v9.0.3)	106	94	96	99	102
Qp(v7.4)	96	92	91	94	97
CDI	70	96	120	136	149
CSA	0.03	0.05	0.06	0.06	0.07
CRI-CAM02UCS	89	89	88	90	94
CRI2012	94	92	92	93	97
MCRI	89	89	90	91	91
Rf	89	87	85	87	93
Rg	106	94	94	97	101
△C*	0.96	−1.02	−0.86	−0.24	0.45
CQI-1	169	173	180	186	191
CQI-2	1,204	1,231	1,634	2,110	2,611
GAI-RA	71	78	86	91	97
GVI	77	81	86	89	91
S_neutral_	0.95	3.45	5.50	6.79	7.37
WS	0.17	0.37	0.55	0.69	0.77
Percent tint	0.68	0.30	0.16	0.55	0.94
DSI	81	86	89	90	94
CDM	−0.24	3.21	5.11	6.04	6.57
MCPI	83	92	102	109	115

CCT: correlated color temperature; (x, y): CIE 1931 chromaticities; (u’, v’): CIE 1976 chromaticity coordinates; Duv: distance from the test chromaticity coordinates to the Planckian locus; CRI: CIE general color rendering index (Nickerson and Jerome, 1965); GAI: gamut area index (Freyssinier and Rea, 2010); CQS: Color Quality Scale (Qa, Qf, Qg, Qp) (Davis and Ohno, 2010); CDI: Color Discrimination Index (Thornton, 1972); CSA: Cone Surface Area (Fotios and Levermore, 1997); CRI-CAM02UCS: Color Rendering Index calculated in CAM02UCS (Luo, 2011); CRI2012: Updated version of CRI (Smet et al., 2013); MCRI: Memory Color Rendering Index (Smet et al., 2010); Rf (color fidelity score) and Rg (color gamut score): IESNA TM-30 metrics (David et al., 2015); △C*: mean chroma shift of CQS (Khanh and Bodrogi, 2016; Khanh et al., 2016a,b); CQI-1 and CQI-2: two of the latest combined metrics named Color Quality Index (Khanh et al., 2016a,b); GAI-RA: the arithmetic mean value of GAI and CRI (Smet et al., 2011; Jost-Boissard et al., 2014); GVI: Gamut Volume Index (Liu et al., 2017b); Sneutral: Degree of neutrality (Kevin et al., 2014); WS: White Sensation (Wang et al., 2015); percent tint: a recently proposed whiteness metric for lighting (Rea and Freyssinier, 2013; Wang et al., 2015); DSI: Daylight Spectrum Index (Acosta, 2017); CDM: color discrimination metric based on meta-analysis (Liu Q. et al., 2020); MCPI, color preference index based on meta-analysis (Huang et al., 2021a).

### 2.3. Lipsticks

Based on sales data on e-commerce platforms, three popular lipstick colors commonly used in China were selected: true red, orange red, and cameo red. For each color type, five lipsticks with different prices and brands were selected. These lipsticks basically represent the most typical lipsticks currently available on the market. To minimize the influence of gloss and texture, all the 15 lipsticks had the same matte texture. All lipsticks were purchased from the official website or flagship store of each brand. Every lipstick was assigned with an identifying number (A0-C4) based on its color, with A representing true red, B representing orange red, and C cameo red.

**Figure 2:**
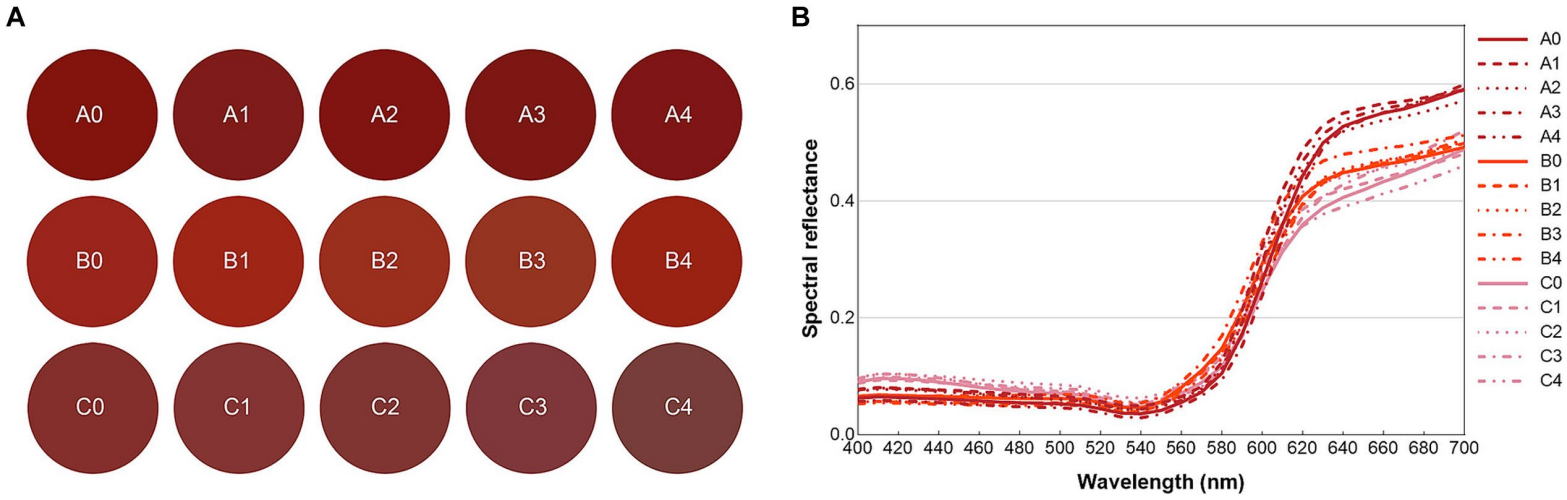
Display of the lipstick colors **(A)** and spectral reflectance of typical colors of the experimental lipsticks **(B)**. A0-A4 are true red, B0-B4 are orange red and C0-C4 are cameo red.

Figure 2 illustrates the simulated color appearance of each lipstick and the spectral reflectance of the experimental lipsticks. The spectral reflectance data were acquired using our formerly proposed spectral imaging system, which achieved accurate spectral reflectance reconstruction based on digital camera responses (RGB values) expansion and pseudo inverse operation (Liang and Wan, 2017).

**Figure 3:**
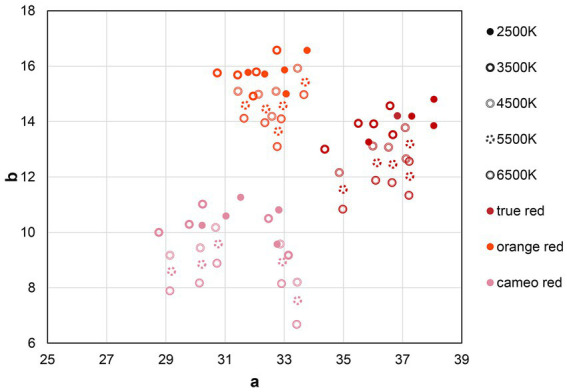
The CAM16-UCS colorimetric coordinates of 15 lipstick colors under five experimental light sources.

Based on the spectral reflectance of the experimental lipsticks and the relative SPDs of the light sources, the colorimetric coordinates of the lipstick colors under the different light sources were calculated in the CAM16-UCS uniform color space (Li et al., 2017). The colors of the lipsticks used in the experiment under different light sources are plotted in Figure 3. The CIE1976 color difference equation (Kuehni, 1976) was used to quantify the color differences. Under each light source, the average ΔE between each two lipsticks of each color group were calculated to represent the within-group color differences, which ranged from 3.25 to 4.89 with an average of 4.00 and a standard deviation of 0.65. For between-group color differences, the mean CAM16-UCS values of the lipstick colors from each color group was calculated. Then the between-group differences under the experimental light sources were computed based on the mean CAM16-UCS values and they ranged from 6.64 to 15.40, with a mean of 11.05 and a standard deviation of 3.42. Since the between-group color differences were much larger than the within-group color differences, in the following analysis only the effect of the three colors types, rather than that of individual lipsticks colors is considered.

### 2.4. Models

**Figure 4:**
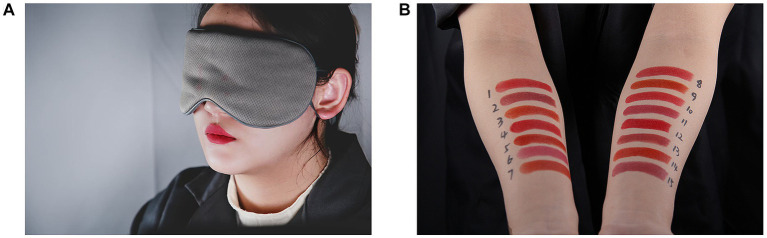
The experimental scenes of the visual test. **(A)** Evaluation for the color of the lips. **(B)** Evaluation for the color stripes on the forearms.

Three female volunteers were selected as models to take turns displaying different lipsticks. Such a setting was not only intended to improve universality, but also to reduce the potential injury to the models’ lips caused by repeated application and removal of lipstick. Prior to the experiment, the models were instructed to clean their faces and apply the appropriate foundation to create similar skin tones. During the experiment, they had to keep their hair tied up, removed any jewelry and they wore an eye mask to cover their face, leaving only the lips exposed, as shown in Figure 4.

### 2.5. Experimental design

The experiment was completed by 60 observers. They were divided into 10 groups with each group consisting of three females and three males. During the experiment, the six observers were instructed to enter the observation area and freely walk around to observe the lipstick colors presented on the model’s lips, or the inside of the forearms, under different light sources. In each experiment group, nine lipsticks (three per color) were selected from 15 to minimize potential lip damage (caused by frequently application and removal) to the model and control the experimental time. Each lipstick was tested an equal number of times. The presentation order of the lipsticks and the experimental light sources was randomized and counterbalanced between successive groups. Additionally, a randomly selected light source was rated twice to quantify the intra-observer variability of each observer.

Each model showed the lip color by wearing a specific lipstick under six light sources (5 testing sources +1 repeat trial) before displaying the color stripes on the forearms under one of the light sources, as shown in Figure 4. This process was repeated for total of nine times in each experimental group. The sequence of color stripes on the forearms, which varied for each model and each experimental group, was randomized and identified by labels from 1 to 15 to enable the observers to record their selections. For the lip color evaluation, a categorical judgment method was employed to assess preference, which adopted a seven-point scale (1, 2, 3, 4, 5, 6, and 7) to represent strongly dislike, moderately dislike, slightly dislike, neutral, slightly like, moderately like and strongly like, respectively. Purchase intention and sexual attractiveness were evaluated similarly. For the color stripes on the forearms, observers were asked to select the three most preferred lipstick colors among all 15 stripes and then assigned them with scores of 3, 2, and 1 according to the order of preference. It is important to note again that, the presentation order of models, lipsticks, and light sources was fully randomized and counterbalanced during the visual tests.

### 2.6. Experimental procedure

Upon arrival, participants were asked to take the Ishihara Test. This was aimed to screen the qualified subjects to ensure they had normal color vision. The experimenter then instructed the qualified participants to put on gray coats to prevent any potential reflections from their clothing. Participants were then required to sign a consent form and complete a general information survey. They were also provided with evaluation forms to record their responses. Subsequently, the ambient room lights were switched off, leaving only the experimental light for illumination.

Prior to the start of the experiment, participants were allowed approximately 5 min to adapt to the experimental lighting condition. During this time, the experimenter provided verbal instructions to the observers (in Chinese) and requested them to close their eyes. Then, a model wearing the eye mask and a specific lipstick entered the experimental area and took a seat. Upon opening their eyes, the observers initiated the observation and rating process, evaluating the model’s lip color along three dimensions: color preference, purchase intention, and sexual attractiveness, using a seven-point rating scale. The experimenter then switched the light sources in a random order, which would be repeated five times until all six light sources had been presented (including a repeated light source for intra-observer variability). Each time the light source was switched, observers were instructed to close their eyes to eliminate any potential influence from the previous lighting condition on their short-term memory. They were then asked to observe the gray background in the lit environment for 30 s for visual adaption. Following the completion of the lip color evaluation under the six light sources, the model displayed 15 color stripes on her forearms, and the observers were asked to select their top three preferred colors under the last light source. This constituted one complete evaluation block for one lipstick, which would be repeated until nine different lipsticks had been assessed. The presentation order of the lipsticks was randomized for each experimental group and each model. The entire experimental procedure lasted 60–80 min.

### 2.7. Data analysis methods

#### 2.7.1. Standardized residual sum of squares values

The standardized residual sum of squares (STRESS, with values that range from 0 to 100) was used to quantify the observer variability (Melgosa et al., 2011). As stated above, to quantify the intra-observer variability, participants were required to rate a randomly selected light source twice without being informed of this. The intra-observer variability was quantified by calculating the STRESS values between repeated ratings and their respective initial ratings. The inter-observer variability was evaluated by calculating the STRESS values between each observer’s ratings and the average ratings of the observers. Lower STRESS values indicate higher data consistency.

#### 2.7.2. Repeated measures analysis of variance

The data obtained from the participants were analyzed using IBM SPSS version 26. A mixed between-within subjects ANOVA was conducted, with five CCT levels, three lipstick colors, and three models as within-subject factors and gender as the between-subject factor, which was repeated three times for the three response scales. In these analyses, the degrees of freedom were adjusted by the method of Greenhouse–Geisser (Greenhouse and Geisser, 1959) if the Sphericity assumption was violated via the Mauchly’s Sphericity test. If a significant main effect or simple effect of an individual factor was determined, *post hoc* comparisons were conducted with Bonferroni adjustment. A probability of *p* = 0.05 was used as significance level for statistical testing.

#### 2.7.3. Pearson correlation coefficients

Pearson correlation coefficients were computed in order to assess the relationship between the average score of each lipstick and its corresponding average STRESS value for inter-observer variability. It was also used to further explore the correlation between the subjective ratings and typical color quality metrics, as well as to demonstrate whether there was a significant correlation between preference ratings for lipstick colors on lips and on forearms. Equally, a probability of *p* = 0.05 was used as significance level for statistical testing.

## 3. Results

### 3.1. Observer variability

**Figure 5:**
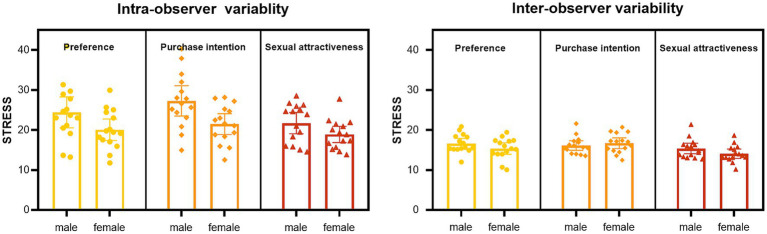
Intra- and inter-observer variability.

To explore gender differences, we calculated observer variability for males and females separately. For the three dimensions, the mean STRESS values of intra-observer variability were 24.40 (male) and 20.05 (female) for color preference, 27.27 (male) and 21.46 (female) for purchase intention, 21.72 (male) and 18.87 (female) for sexual attractiveness. For inter-observer variability, the mean STRESS values were 16.62 (male) and 15.37 (female) for color preference, 16.12 (male) and 16.69 (female) for purchase intention, 15.36 (male) and 14.07 (female) for sexual attractiveness. The distribution of the STRESS values for males and females are shown in Figure 5.

### 3.2. Overall results

**Figure 6:**
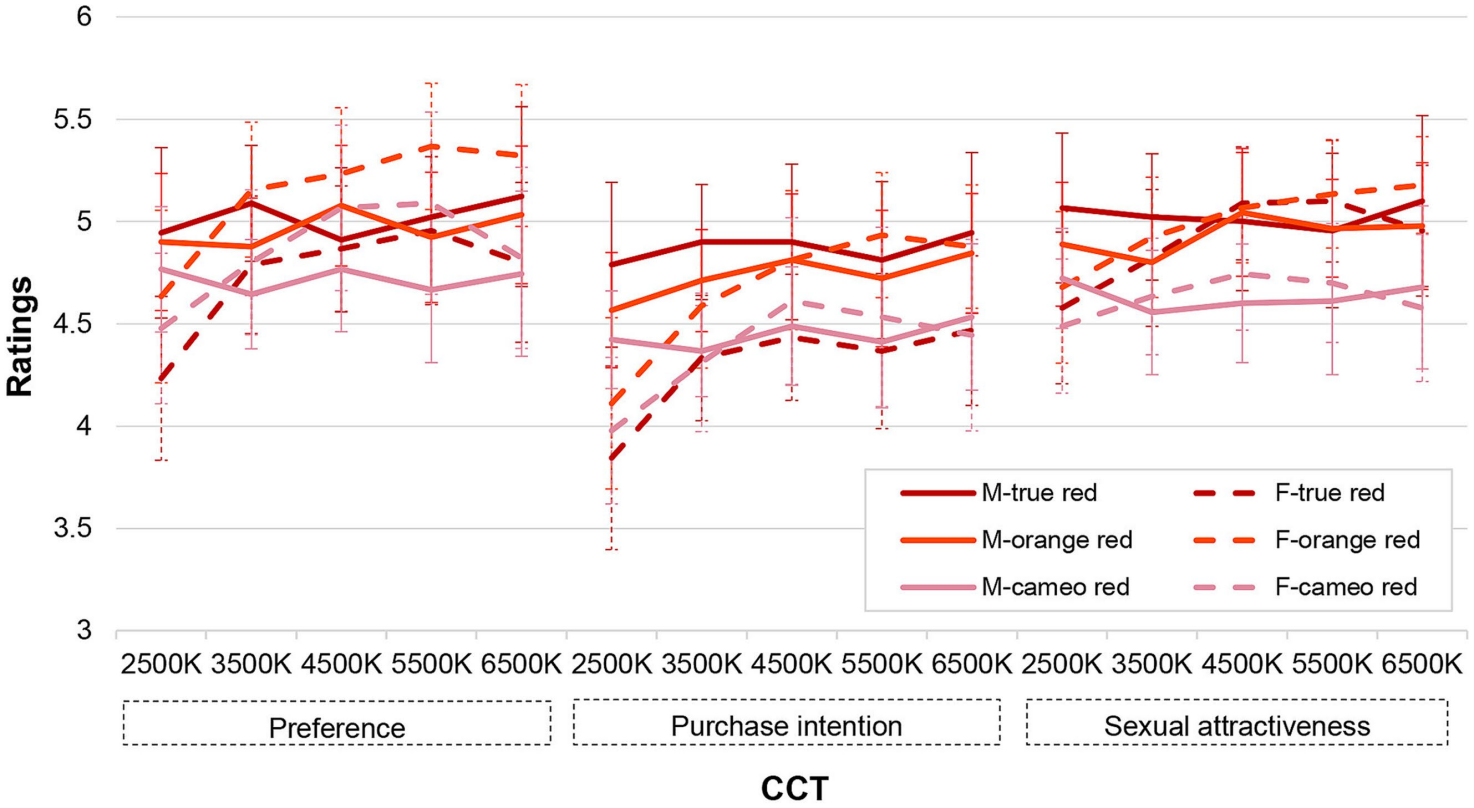
Mean observer ratings for three colors in three response scales under five experimental light sources. “M” represents the male observers and “F” represents the female observers. The error bars denote the 95% confidence interval.

Figure 6 illustrates the average evaluation ratings of the three lipstick colors across five light sources with varying CCTs for three response scales. From these charts, a notable disparity between male and female observers was found in the trends of all three scales with different CCTs. Specifically, male observers’ ratings exhibited smaller discrepancy, whereas females’ ratings increased and subsequently decreased with CCTs from 2,500 K to 6,500 K. The optimal lipstick color could not be easily identified as male and female observers showed a different attitude for different lipstick colors.

Table 2 summarizes the overall results of repeated measures ANOVA, indicating that for preference and purchase intention, the effect of CCT was statistically significant with the largest effect size (*η*^2^) of 0.102 and 0.132. The interaction between CCT and gender was also significant. While the effect of lipstick color was significant only in the dimension of sexual attractiveness. Although the model variable had a significant effect on subjects’ evaluations for preference and purchase intention, no significant interactions were found between model and other factors, implying that model only played an independent role and did not interact with other experimental variables. Therefore, the effect of models would not be reported in the following results.

**Table 2:** Statistical significance and effect size of the effect of the independent variables (CCT, lipstick color, model and gender) on the ratings of preference, purchase intention and sexual attractiveness.

Scale	Independent variable or interaction	Sum of square	df	Mean square	F	Sig.	*η*^2^
Preference	**CCT**	**14.719**	**2.133**	**6.9**	**6.594**	**0.002**	**0.102**
	Color	11.161	2	5.58	2.739	0.069	0.045
	**Model**	**22.547**	**2**	**11.274**	**4.916**	**0.009**	**0.078**
	Gender	0.015	1	0.015	0.002	0.962	0
	CCT*Color	2.12	5.041	0.42	0.928	0.464	0.016
	CCT*Gender	**12.151**	**2.133**	**5.696**	**5.443**	**0.005**	**0.086**
	Color*Gender	10.008	2	5.004	2.456	0.09	0.041
Purchase intention	**CCT**	**19.813**	**2.233**	**8.873**	**8.833**	**<0.0001**	**0.132**
	Color	12.547	2	6.274	2.954	0.056	0.048
	**Model**	**20.229**	**2**	**10.114**	**4.401**	**0.014**	**0.071**
	Gender	12.8	1	12.8	2.201	0.143	0.037
	CCT*Color	1.811	5.507	0.329	0.798	0.562	0.014
	CCT*Gender	**9.058**	**2.233**	**4.056**	**4.038**	**0.016**	**0.065**
	Color*Gender	**13.119**	**2**	**6.559**	**3.088**	**0.049**	**0.051**
Sexual attractiveness	CCT	5.198	2.146	2.423	2.496	0.083	0.041
	**Color**	**22.596**	**2**	**11.298**	**8.342**	**<0.0001**	**0.126**
	Model	1.156	2	0.578	0.31	0.734	0.005
	Gender	0.104	1	0.104	0.019	0.89	0
	CCT*Color	1.556	6.185	0.252	0.822	0.556	0.014
	CCT*Gender	5.388	2.146	2.511	2.588	0.075	0.043
	Color*Gender	1.248	2	0.624	0.461	0.632	0.008

Bold font denotes statistical significance (*p* < 0.05).

### 3.3. Impact of light source

Since the effects of CCT were significant on preference and purchase intention, the *post hoc* tests were conducted. The results indicated that the preference and purchase intention ratings for a CCT of 2,500 K were significantly lower than those for the other four CCTs. However, there was no significant difference between any two light sources for sexual attractiveness.

To further examine the interactions between CCT and gender, simple effect tests were also conducted. The results indicated that the effect of light sources was significant for female observers, but not for male observers (Female: *p* < 0.001, *η*^2^ = 0.368 for preference, *p* = 0.001, *η*^2^ = 0.276 for purchase intention, ratings under 2,500 K were significantly lower than the other four light sources. Male: *p* = 0.620, *η*^2^ = 0.046 for preference, *p* = 0.498, *η*^2^ = 0.058 for purchase intention). Furthermore, CCT had a significant effect on females’ ratings of sexual attractiveness with an effect size smaller than the other two dimensions (*p* = 0.021, *η*^2^ = 0.186, the ratings under 2,500 K were significantly lower than those under 4,500 K). Similarly, the simple effect of CCT was not significant for males (*p* = 0.497, *η*^2^ = 0.058).

### 3.4. Impact of lipstick color

The effect of lipstick color was found to be not significant in terms of preference (*p* = 0.069, *η*^2^ = 0.045) and purchase intention (*p* = 0.056, *η*^2^ = 0.048), but to be significant for sexual attractiveness (*p* < 0.001, *η*^2^ = 0.126). The *post hoc* tests revealed that the ratings of sexual attractiveness for cameo red was significantly lower than that for true red (*p* = 0.003) and orange red (*p* = 0.003), while there were no significant differences (*p* > 0.05) among the other two dimensions.

The interaction between lipstick color and gender was also further explored. For preference, lipstick color had a significant effect on females’ ratings (*p* = 0.02, *η*^2^ = 0.128), with the *post hoc* test revealing that the ratings for true red were significantly lower than orange red (*p* = 0.018). For purchase intention, lipstick color had a significant impact on males’ ratings (*p* = 0.029, *η*^2^ = 0.115), with the ratings for true red almost significantly higher than cameo red (*p* = 0.065). In term of sexual attractiveness, lipstick color had a significant impact for both male (*p* = 0.021, *η*^2^ = 0.127) and female observers (*p* = 0.034, *η*^2^ = 0.112), with males giving significantly higher ratings for true red than for cameo red (*p* = 0.019), and females giving lower ratings for cameo red than for orange red (*p* = 0.031). Overall, considering the average ratings of lipstick color as shown in Figure 6, male observers’ ratings were highly consistent across all three dimensions with, in general, the highest score for the true red and the lowest score for the cameo red. Female observers maintained the highest ratings for orange red but exhibited more variations in the lowest ratings.

### 3.5. The evaluation of color stripes on the forearm

**Figure 7:**
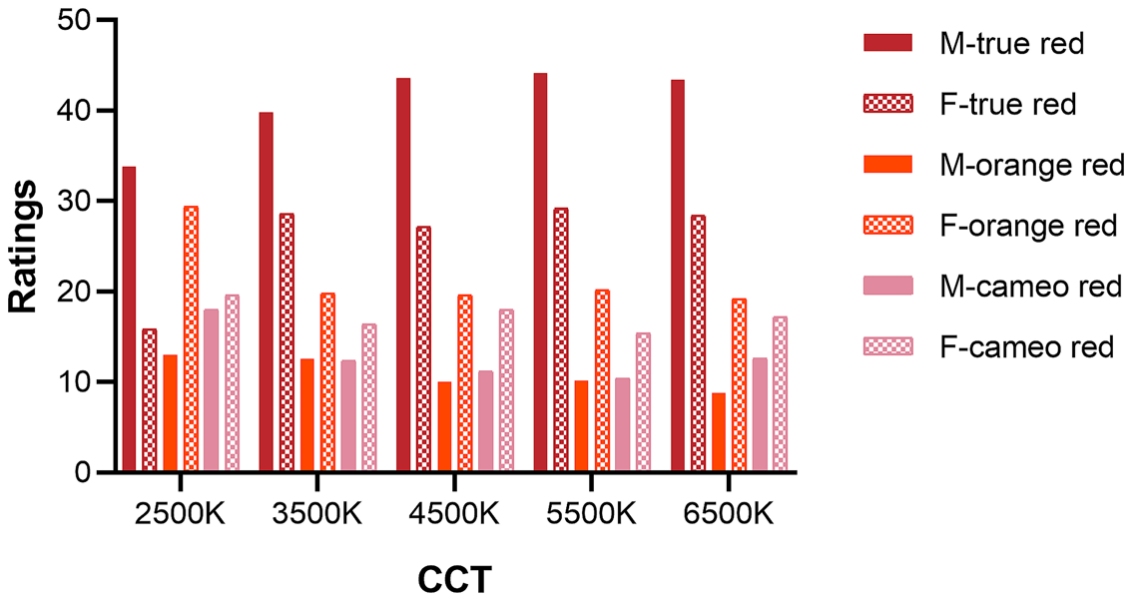
Male and female observers’ overall preference scores of each color type under five light sources.

Figure 7 illustrates the total scores for each lipstick color obtained by observing the color stripes on the models’ forearms. Generally speaking, both males and females rated the true red color with the highest scores, followed by orange red, and then cameo red. However, males show a much greater preference for true red compared to other colors, while the attitude of females toward different lipstick colors was not so pronounced. Furthermore, under the 2,500 K light source, females exhibited a noticeable decrease in preference for true red compared to other light sources.

The correlation between the preference for lip colors and color stripes on the forearms was explored using the Pearson correlation coefficient. Table 3 presents the results for male and female observers under different light sources, indicating that there was no significant correlation between the preference ratings judged from the lips and forearms.

**Table 3:** Pearson’s *r* and *p*-values for different presentation (i.e., on lips or on forearms) of lipstick colors.

	Overall		Male		Female	
	*r*	*p*	*r*	*p*	*r*	*p*
Overall	0.15	0.59	0.35	0.20	0.32	0.24
2,500 K	0.19	0.51	0.13	0.65	0.26	0.35
3,500 K	0.19	0.50	0.34	0.22	0.29	0.30
4,500 K	0.06	0.82	0.14	0.62	0.21	0.44
5,500 K	0.21	0.45	0.57	0.03	0.07	0.81
6,500 K	0.07	0.80	0.26	0.36	0.17	0.55

## 4. Discussion

The observer variability results in Section 3.1 indicated that females exhibited greater stability in individual ratings when repeating evaluations, shown by the values of intra-STRESS for females being lower than those for males. This result corroborated previous findings by Hurlbert and Ling (2007), which demonstrated that the preference curves of individual females in the “color picking” task with 208 participants remained more consistent than males over time. Such gender difference can be partially attributed to evolutionary and social factors that have influenced females’ specialized abilities in gathering, which require higher sensitivity in color vision, particularly with regard to distinguishing ripe red and orange fruits from green foliage as posited by the hunter-gatherer theory (Silverman and Eals, 1992). Additionally, as demonstrated by Bimler et al. (2004), differential socialization patterns may have contributed to females’ greater color awareness. Moreover, physiological distinctions in color vision between genders have also been documented. Studies indicate that inherent variations exist in retinal physiology and visual cortical processing between males and females (Vanston and Strother, 2017). The genes on the X chromosome have been found to determine the spectral sensitivities of many photoreceptors in the retina (Neitz and Neitz, 2011). Some studies also demonstrated that women are more responsive to red and yellow hues (McGuinness and Lewis, 1976; Hoyenga and Wallace, 1979). Interestingly, these colors were the primary shades of the lipsticks employed in the current experiment. Thus, it is possible that females are more adept at discerning color differences, leading to more stable ratings in repeated evaluations.

Furthermore, the analysis revealed that for every response scale, the average score of each lipstick and its corresponding average STRESS value for inter-observer variability were always negatively correlated, with Pearson correlation coefficients ranging from −0.60 and −0.85. This finding suggested that observers exhibited greater consistency in their responses when they preferred a certain lipstick color, which was in line with our previously-reported results (Huang et al., 2017; Liu et al., 2017a; Deng et al., 2022).

### 4.1. Impact of lighting and interaction with gender difference

In terms of the effect of light sources on observer evaluations, the ratings under 2,500 K were significantly lower than the other four light sources in the dimension of preference and purchase intention. For CCTs from 3,500 K to 6,500 K, there was no significant difference and the highest average rating of preference were obtained for the CCT of 5,500 K. Interestingly, this was consistent with our earlier research, which suggested that the optimal CCT with the highest subjective preference score was 5,500 K (Huang et al., 2018). It may corroborate our previous experimental results that under the investigation and comparison of several contextual factors including spectral power distribution of light, lighting application, observers’ personal color preference, regional cultural difference and gender difference, the influence of light sources was consistent and had a dominate effect on color preference when CCTs differ (Huang et al., 2017). This was also strengthened by the largest effect size (*η*^2^ = 0.102) of main effect of CCT.

Our previous research found that there was a strong correlation between the degree of neutrality, color preference, and color discrimination (Huang et al., 2019a,b; Deng et al., 2022). Therefore, three color quality metrics were selected to examine the former findings. The degree of neutrality (S_neutral_) metric (Kevin et al., 2014), an empirical metric modeled by a bivariate Gaussian function, was uesd to predict the degree of neutrality of lighting stimulus viewed under dark adapted conditions by an average observe. The color discrimination metric (CDM) (Liu Q. et al., 2020), building on human visual adaptation to the chromaticities of natural light as well as the large range of the SPDs of modern light sources, was adopted for quantifying the color discrimination capability of white light sources. The color preference index based on meta-analysis (MCPI) (Huang et al., 2021a) was a combination of an absolute gamut-based metric and a fidelity-based metric, which was derived by fitting the large psychophysical dataset and was used for color preference predictions. Notably, the advantages of those three metrics beyond other existing metrics have been verified by our previous work using meta-analysis approaches (Liu Q. et al., 2020; Huang et al., 2019b, 2021a). In this study, we explored the correlation between the values of S_neutral_, CDM, MCPI of the experimental light sources and mean subjective ratings of observers. Due to the significant interaction between gender and light sources, the Pearson correlation coefficients for male and female ratings were calculated separately, as shown in Table 4.

**Table 4:** Pearson correlation coefficient between subjective ratings and typical color quality metric values.

		Female			Male		
		S_neutral_	CDM	MCPI	S_nuetral_	CDM	MCPI
Color preference	*r*	0.705**	0.740**	0.643*	0.159	0.153	0.176
	*p*	**0.003**	**0.002**	**0.010**	0.570	0.586	0.530
Purchase intention	*r*	0.762**	0.792***	0.714**	0.257	0.263	0.261
	*p*	**0.001**	**<0.001**	**0.003**	0.355	0.344	0.348
Sexual attractiveness	*r*	0.599*	0.617*	0.554*	0.047	0.024	0.071
	*p*	**0.018**	**0.014**	**0.032**	0.869	0.932	0.801

Bold font denotes statistical significance, *p < 0.05, **p < 0.01, ***p < 0.001.

The results indicated that there were remarkable gender differences in all three dimensions. For female observers, the correlations in three dimensions were all significant, and were stronger in preference and purchase intention, indicating that the impact of light sources on these two dimensions was greater than that on sexual attractiveness. This suggested that females had a higher preference and purchase intention under light sources closer to neutral white, and with higher color preference and discrimination quality. However, for male observers, the correlations were not significant and generally weaker, especially for sexual attractiveness. This may be because the sexual attractiveness was closely related to the red effect, which was associated with lipstick color and almost unaffected by the light sources. These results were consistent with the significant interaction between light sources and gender, indicating that light sources primarily affect color perception in female observers rather than male observers.

One possible explanation for the above findings was that lipstick was a familiar object for females but not so much for males. Previous research has reported that the familiarity of experimental objects influences preference rating range (Huang et al., 2017), i.e., the rating range for familiar objects was usually larger than that for unfamiliar objects since, usually, people had a preconceived idea about the colors of objects they were familiar with. Therefore, when the experimental objects were familiar, the rating differences among light sources were more likely to be evident. In addition, the gender differences may be rooted in physiological disparities related to color vision in males and females. Research has demonstrated that women have higher sensitivity (Rodríguez-Carmona et al., 2008) and better color discrimination abilities compared to men (Vanston and Strother, 2017). Some researchers believe that the gender difference in color perception could be attributed to “*a sexual dimorphism in the gene that encodes the photopigment of the long-wavelength sensitive cones in the retina, manifest in a different frequency of expression in men and women*” (Pardo et al., 2007).

Additionally, gender differences have always been an interesting focus in psychophysical experiments of color perception. In the current study, an interaction between different CCTs and gender was found in terms of lipstick color preference, primarily at the light source with a CCT of 2,500 K. For color preference ratings under 2,500 K, the gender difference is close to significance, with *p* = 0.066. However, after revisiting our former psychophysical data (Huang et al., 2017, 2018, 2019a, 2020a,b, 2021b; Chen et al., 2020; Liu Y. et al., 2020; Wang et al., 2020) on color preference of lighting with various experimental objects, significant gender difference was only observed in the work that related to blue jeans (Liu Y. et al., 2020) under light sources with positive Duv values. We find that the two research studies that revealed significant gender difference had one thing in common: the experimental light sources were of yellowish (low CCT in this study) or greenish (positive Duvs in Liu Y. et al., 2020) colors and the observed objects were monochromatic (i.e., Jeans or lipsticks). This suggests that gender differences may be more evident for such a specific condition, indicating the need for further exploration.

### 4.2. Impact of lipstick color and the interaction with gender difference

As reported in Section 3.4, the impact of lipstick color on sexual attractiveness was particularly prominent. This finding supports the theory regarding the specific association between red color and sexual attractiveness and also provides validation for our hypothesis that moderate variations in different shades of red lipsticks can lead to distinct perceptual and psychological effects. Throughout human evolution, sexual attractiveness has played a crucial role in mate selection, with males exhibiting a preference for specific features that indicate high mate value in potential female partners (Symons, 1995). Lip shape and color, which serve as indicators of youth and fertility (Fink and Neave, 2005), may offer important cues for evaluating partner value.

**Figure 8:**
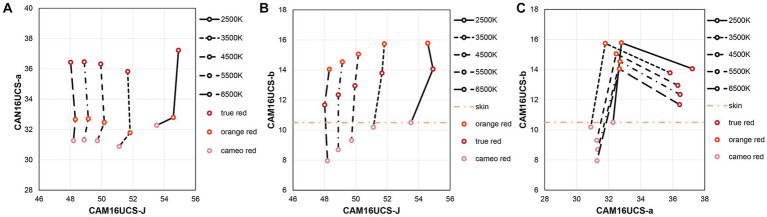
Coordinates of lipstick colors in CAM 16-UCS under different light sources: **(A)** J-a plot, **(B)** J-b plot, and **(C)** a-b plot.

Our findings revealed that men generally favored true red. This was consistent with previous research indicating that red lipstick was more effective in enhancing women’s attractiveness to men compared to pink and brown shades (Guéguen, 2012b; Guéguen and Jacob, 2012b). To further explore the color appearance of different lipsticks used in the experiment, their colors under five light sources were plotted in CAM16-UCS, as shown in Figure 8. The results show that the values on the a-axis for true red are higher than those for the other two colors, implying that true red lipstick increases redness of the lips to a greater extent. In studies conducted by Stephen and McKeegan (2010), it was found that as the redness of lips increased, there was a corresponding increase in contrast between the lip color and skin tone, which can enhance femininity by reflecting higher levels of estrogen. Moreover, as women age, the red-green contrast between their lips and surrounding skin tended to decrease due to changes in blood flow (Kim et al., 2019). Therefore, the true red shade with higher values on the a-axis may denote a woman’s higher estrogen levels and younger appearance. They were vital factors related to fertility and served as important cues for males in assessing potential mate values. Additionally, studies have demonstrated that the color red can enhance the perceived sexual receptivity and intent of women (Pazda et al., 2012, 2023). Compared to orange red and cameo red shades, true red may possess more distinctive arousing properties, leading to higher ratings from male observers.

The cameo red lipstick received the lowest ratings in all three dimensions for males. In Figure 8, it is evident that the values on the b-axis of cameo red are the lowest, resulting in a negative yellowness contrast against the skin tone. This suggests that the “blueness” of the lips was more prominent when wearing cameo red lipstick. However, blue lips are a sign of cyanosis (lack of blood oxygen), which is associated with respiratory and cardiac illnesses (Ponsonby et al., 1997). These features are actually indicative of poor physiological health and low mate value (Fink and Neave, 2005). As mentioned earlier, males have evolved a psychological mechanism to selectively detect and respond to specific features of female bodies that are relevant to mate selection (Symons, 1995). Thus, the association between bluish lips and low mate values may lead to the lowest ratings of cameo red for male observers.

Furthermore, it is noteworthy that females’ ratings for true red lipstick were the lowest in the dimension of preference and purchase intention. However, in terms of sexual attractiveness, consistent with males, the true red turned out to be the first choice for females as well. We hypothesized that females were able to perceive the unique association between true red and sexual attractiveness. Similarly, findings have been reported by Elliot et al.: when given the option to choose their shirt color based on the conversation partner, women showed a clear preference for wearing red shirts when interacting with attractive men, compared to unattractive men or attractive women (Elliot et al., 2013). It was suggested that red not only functions as a sexual signal to be received by men, but can also be used as a sexual signal to be sent by women. Additionally, Gomes’s study on women’s views of dress exposure found a strong correlation between the Attractiveness (physical attractiveness perceived by themselves) and the Partner’s Attraction (the extent to which women felt that their male partner would perceive the woman as attractive) (Gomes, 2017), such results indicate that women’s ratings were consistent with the hypothetical males’ ratings. As we believe, it is highly probable that female observers in our experiment imagined a male subject rating the sexual attractiveness of the lipstick color and the imagined subject was consistent with the actual male participants, leading to similar results between male and female observers.

### 4.3. Inconsistency between color on lip and color on forearm

A clear finding in the evaluation of preference on color stripe on the forearm was that both men and women preferred true red over orange red and cameo red. The color appearance attributes of the lipstick colors on the forearm were also calculated in CAM16-UCS (Li et al., 2017), including lightness (J), hue angle (h), chroma (C), and colorfulness (M). It was found that the observer’s preference ratings had strong correlation with the chroma (C) and the colorfulness (M), with Pearson correlation coefficient equaled to 0.724 and 0.721, respectively. Such a finding was aligned with previous research that an increase in chroma significantly impacted color preference (Guilford, 1934; Jost-Boissard et al., 2014; Wei et al., 2014; Lin et al., 2015).

Another noteworthy finding was that although females generally preferred true red, their preference for true red remarkably decreased with a CCT of 2,500 K. This result was consistent with the above findings in lip color evaluation, where female participants’ ratings for true red under 2,500 K were significantly lower than other lighting conditions. This suggests that when displaying cosmetics associated with true red, the use of light sources with lower CCT may not be wise for female consumers.

Most importantly, there was no significant correlation between preference ratings for lipstick color presented on lips and forearms. This suggested that observers’ perception and subjective evaluations toward the same lipstick color were different when presented in different ways. Such a result could be for two reasons. First, lips have distinct features and social-cultural meanings that are fundamental to the evaluation of lipstick shades, while forearm stripes are merely ordinary swatches. Lips are a focal point of facial beauty, with pouty and full lips symbolizing youth, attractiveness, and sexuality (Fink and Neave, 2005; DeJoseph et al., 2018). Feminist theory also suggests that lipstick application behavior is relevant to the extension of women’s right to freedom and liberation (Gurrieri and Drenten, 2019) and represents sexual autonomy and desire (Gill, 2003). Thus, evaluating lipsticks on lips may be more intricate than on the forearm, and the different evaluation contexts and observation patterns lead to distinct perceptual and psychological processes among observers. Second, the natural color of the lips and the skin tone on the inner forearm are inherently different, resulting in different color appearances even when applying the same lipstick. This finding revealed that applying the lipsticks on the forearm to select one’s favorite or most suitable lipstick color for daily makeup is not a reliable method, which, however, is precisely the current misconception of many people when conducting color-related evaluations.

## 5. Conclusion

In this study, a psychophysical experiment was conducted to investigate the impact of light source and lipstick color, as well as the gender difference, upon the evaluation for lipstick application. Our results indicated that light sources have a significant impact on preference and purchase intention, while lipstick color has a significant impact on sexual attractiveness. The significant interactions between these two variables and gender support the notion that females are more sensitive to light sources and the true red lipstick shade maximizes the red effect to male observers. In addition, there is no correlation between the preference ratings of lipstick color on the lips and on the forearm, which negated the effectiveness of this commonly used color comparison method. Overall, the findings from our study offer insights into the perception of males and females regarding lipstick colors and highlight the importance of lighting for the lipstick display for female consumers. Considering the effect of lighting, it is suggested that the light sources with low CCT should be avoided in lipstick purchasing scenarios. Meanwhile, as discussed in Section 4.1, for future research targeting gender differences in color perception, it would be interesting to investigate the color preference judgments of males and females for monochromatic objects.

## Data availability statement

The raw data supporting the conclusions of this article will be made available by the authors, without undue reservation.

## Ethics statement

The studies involving humans were approved by the Institutional Review Board (IRB) of Wuhan University. The studies were conducted in accordance with the local legislation and institutional requirements. The participants provided their written informed consent to participate in this study. Written informed consent was obtained from the individual(s) for the publication of any potentially identifiable images or data included in this article.

## Author contributions

BL-T: Methodology, Writing – original draft, Data curation, Formal Analysis. HW-G: Data curation, Formal analysis, Methodology, Writing – original draft. ZY-C: Writing – review & editing. XY: Investigation, Writing – original draft. MP: Writing – review & editing. JY: Data curation, Formal analysis, Writing – original draft. FY: Writing – review & editing, Project administration, Supervision. QL: Writing – review & editing, Project administration, Supervision, Conceptualization, Funding acquisition, Methodology, Resources, Writing – original draft.
